# The nutritional quality of a fresh orange pulp-wheat straw mixture ensiled with either sugar beet pulp, wheat bran, or urea compared to corn silage (*Zea mays*) in sheep

**DOI:** 10.1093/tas/txad017

**Published:** 2023-02-04

**Authors:** Ali Dadashi, Yousef Rouzbehan, Hassan Fazaeli, Javad Rezaei

**Affiliations:** Department of Animal Science, Faculty of Agriculture, Tarbiat Modares University, Tehran, Iran; Department of Animal Science, Faculty of Agriculture, Tarbiat Modares University, Tehran, Iran; Animal Science Research Institute of Iran, Agricultural Research, Education and Extension Organization (AREEO), Karaj, Iran; Department of Animal Science, Faculty of Agriculture, Tarbiat Modares University, Tehran, Iran

**Keywords:** ammonia nitrogen, orange peel silage, rumen variables, sheep, wheat bran

## Abstract

This study compares corn silage (CS) with an orange pulp-wheat straw mixture (OW) ensiled with either sugar beet pulp (SBP), wheat bran (WB), or urea in terms of intake, chemical composition, phenolic compounds, silage fermentation characteristics, digestibility, in vivo rumen variables and biochemical blood variables in 48 Shall male sheep, and in vitro methane (CH_4_) production. In addition to CS, five other silages: OW (i.e., 87.5% fresh orange pulp + 12.5% wheat straw); OWU (OW + 1% urea); OWS (87.5% fresh orange pulp + 8.6% wheat straw + 3.9% SBP); OWSU (87.5% fresh orange pulp + 8.6% wheat straw + 1% urea + 3.9% SBP); and OWB (87.5% fresh orange pulp + 8.6% wheat straw + 3.9% SBP) were ensiled for 90 days. All diets, which contained a mineral-vitamin premix (10 g/kg of dry matter [DM]), were each randomly assigned to five sheep (live weight 40 ± 2.5 kg) using a completely randomized design, and the SAS software MIXED method was used for data analysis. Among all silages, OWU and OWSU had the highest (*P* < 0.01) ammonia-N concentration, but there were no differences in other fermentation characteristics. Animals fed on the CS diet had higher DM intake (*P* = 0.01) and DM (*P* = 0.01), organic matter (*P* = 0.01), and neutral detergent fiber (*P* = 0.02) digestibilities compared with other diets. However, sheep receiving OWU and OWSU diets had higher (*P* < 0.01) crude protein digestibility than those fed on other diets. The OWU and OWSU-fed sheep had the highest (*P* = 0.04) ruminal ammonia-N concentration. Sheep fed on CS had higher (*P* = 0.03) ruminal total short-chain fatty acids, acetate concentration (*P* = 0.02), total protozoa (*P* < 0.01), and cellulolytic bacteria numbers (*P* < 0.01), but had a lower (*P* = 0.03) propionate concentration compared with the other sheep. In vitro CH_4_ production was higher (*P* = 0.01) with the CS diet compared to the orange pulp diets. Estimated microbial protein supply was lower (*P* = 0.05) with CS compared to all orange silages. In conclusion, the variation in the nutritive quality among the OWS, OWSU, and OWB is relatively small, and the OWB, which is most comparable to CS, was judged to be nutritionally the best among the diets.

## Teaser

• Ensiled orange pulp produced good-quality silage that compared with well corn silage.

• The nutrient digestibilities of ensiled orange pulp diets are comparable to corn silage, and it has no adverse effects on ruminal and blood variables.

## INTRODUCTION

Climate change and drought have led to shortages in animal feedstuffs in many parts of the world, which has encouraged the use of agricultural byproducts such as orange pulp (OP) in ruminant rations. The OP is the co-product of orange juice production consisting of membranes, peels, residual pulp, and seeds and represents between 49% and 69% of the wet weight of processed fruit (Leiva et al., 2000). Using OP as an animal feedstuff is a way of recycling that which otherwise can cause environmental pollution (Huber, 1980), and reducing feed costs (Bampidis and Robinson, 2006). Global orange production was 71.4 million t in 2013 (FAO, 2014) producing 20 million t of OP (Osborne and Stalker, 2013). The OP has been used as a metabolizable energy (ME) source (12.8 MJ/kg dry matter [DM]) in ruminant feed and includes abundant pectin levels (223 g/kg of DM), which is fermented in the rumen without affecting pH (Bampidis and Robinson, 2006). The high moisture content of fresh OP (800 g/kg fresh weight, Zema et al., 2018) makes preservation in the form of silage difficult without the addition of other materials to increase the DM content (Volanis et al., 2004). A variety of feedstuffs including wheat straw, dry sugar beet pulp (SB), and wheat bran (WB), have been investigated as additives. Wheat straw can be effective in wet material silages, ensuring adequate substrate supply throughout the fermentation process and minimizing undesirable fermentation (Scerra et al., 2001). Rouzbehan et al. (1996) included sugar beet pellets as an absorbent in big bale grass silage, where they observed an increase in water retention by the sugar beet and raised levels of water-soluble carbohydrate (WSC) and lactic acid in the silage. Zanine et al. (2006), also, reported that WB could be used in silages as an absorbent additive, minimizing silage effluent losses and adding nutritional value.

Rihani et al. (1993) used urea addition for the N enrichment of high-energy, low-N co-products, especially those with high pectin levels. The latter compounds readily bind with ammonia to form polygalacturonic acid amides and other ammonium compounds that degrade more slowly in the rumen than urea (Kowalczyk, 1976), and this may lead to a more consistent supply of ammonia N for increasing fibrolytic microbial activity and the microbial protein synthesis (Guo et al., 2022).

Corn silage (CS) is the predominant forage used in ruminant diets worldwide, so was used as a control in this study investigating nutritional quality, chemical composition, phenolic compound levels, and silage fermentation characteristics along with five OP silages. These silages were also compared in terms of dietary intake, digestibility, in vivo rumen variables, and blood biochemistry when fed to sheep.

There is limited data on feeding OP ensiled with urea, SB, and WB, as a complete diet, and its ability to provide the maximum ruminal microbial and sheep requirements. Hence, the nutritional quality of an OP-wheat straw mixture ensiled with either SP, WB, or urea compared with CS (*Zea mays*) in sheep rations was evaluated.

## MATERIALS AND METHODS

The animal husbandry, as well as the manner of rumen fluid collection, in this work, were approved by the University of Tarbiat Modares (Proposal approved 9730091003, 2018).

### Silage Preparation, Animals, and Management

Corn seeds (*Z. mays*, var. hybrid SC 704) were obtained from the Seed and Plant Research Improvement Institute (Karaj, Iran), and were harvested 45 days after sowing when the corn had reached the milk stage (Growth stage BCH-75; Lancashire et al., 1991). The corn forage was wilted for 30 h, cut into approximately 2–4 cm lengths, a mixture homogeneously placed into 10-L double layer plastic bags (four replicates), and a vacuum applied before the bags were sealed then left to ensile for 90 days. OP (*Citrus sinensis*), came unprocessed from the Chaboksar factory (Guilan province, Iran), then was coarsely chopped using a modified maize chopping machine. Wheat straw was harvested at full maturity and cut into 3–5 cm lengths. In addition to CS, the other five silages were OW (87.5% fresh orange pulp + 12.5% wheat straw); OWU (OW + 1% urea); OWS (87.5% fresh orange pulp + 8.6% wheat straw + 3.9% SBP); OWSU (87.5% fresh orange pulp + 8.6% wheat straw + 1% urea + 3.9% SBP); and OWB (87.5% fresh orange pulp + 8.6% wheat straw + 3.9% SBP). They were ensiled in 10-L double-layer plastic bags (5 replicates, totaling 240 bags) for 90 days. For further analysis, samples from all silages (3 kg) were stored at −20 °C (Table 1).

**Table 1.** Chemical composition, mean ±SD (standard deviation), of fresh orang pulp, wheat straw, sugar beet pulp, and wheat bran (g/kg DM or as stated) (n = 5)

Item	Fresh orange pulp	Wheat straw	Sugar beet pulp	Wheat bran
DM, g/kg fresh weight	149 ± 2.01	880 ± 1.98	870 ± 3.44	870 ± 2.82
Ash	60.1 ± 4.48	68.5 ± 1.41	87.9 ± 1.32	65.2 ± 2.50
CP	61.0 ± 3.27	36.0 ± 0.28	74.2 ± 2.61	128.1 ± 3.02
NDF	225 ± 3.50	830 ± 5.06	446 ± 4.22	450 ± 4.54
ADF	162 ± 2.47	549 ± 2.00	267 ± 1.80	135 ± 1.55
ADL	27.1 ± 2.53	100.2 ± 2.05	39.8 ± 0.92	35.5 ± 1.62
Pectin	310 ± 2.16	ND	ND	ND
WSC	50.2 ± 3.53	11.1 ± 0.50	84.3 ± 3.26	81.3 ± 2.20
EE	24.1 ± 1.51	11.5 ± 0.65	5.4 ± 0.12	39.5 ± 1.77
Starch	80.5 ± 1.48	11.8 ± 0.94	65.4 ± 1.44	179.2 ± 3.13
Non-fiber carbohydrates	630.4 ± 4.62	54.0 ± 1.52	386.7 ± 5.01	317.0 ± 3.05
ME, MJ/kg DM	10.0 ± 1.24	6.3 ± 1.11	10.1 ± 1.50	11.1 ± 1.18

Non-fiber carbohydrates calculated as: 1000 – (NDF g/kg + CP g/kg + ether extract g/kg + ash g/kg); ME, calculated as: 2.20 + 0.136 × gas production + 0.057 × CP + 0.0029 × CP^2^ (Menke et al., 1979).

ND, not determined; ADF, acid detergent fiber.

Forty-eight Shall male sheep (body weight [BW] 40 ± 2.2 kg) were assessed for 40 days. They were penned individually (1.5 m × 1.5 m pens) on woodchips and offered the rations for 33 days. The sheep were then placed in metabolism crates (0.6 m × 1.3 m) for the final 7 days to allow for the daily collection of orts, feces, and urine (2 days for crate adaptation and five sampling days). A premix of mineral-vitamin (10 g/kg of DM) was added to each silage diet. The diets were balanced for the maintenance of male sheep according to the recommendations of NRC (2007) and fed twice daily at 08.00 and 16.00 as a total mixed ration. The animals had continuous access to fresh water and water intake was determined daily. At the beginning and end of the experiment, BWs were noted to confirm the adequacy of the maintenance diet.

### Analytical Procedures and Fermentation Characteristics of Silage

For 48 h at 60 ^°^C, silage samples were dried for determination of the DM, and milled using a 1-mm sieve to be used for further analysis. Nitrogen (N) levels were measured using the procedure 990.03 of AOAC (2012) and crude protein (CP) was obtained by multiplying by 6.25. An AOAC (2012) method was used to measure the ash content. The concentration of ether extract (EE) was measured using AOAC (2012) procedure method 2003.05. The determination of ash-free neutral detergent fiber (NDF) was carried out using the Van Soest et al. (1991) method. Method no. 973 (AOAC, 2012) was used to measure ash-free acid detergent fiber and expressed exclusive residual ash. Lignin analysis was carried out according to AOAC (2012); method 973.18. The WSC level was measured by the anthrone reaction test, using a spectrophotometer to measure the extract absorbance (MAFF, 1986).

Silage pH was determined by the method of Faithfull (2002). Silage was compressed manually to produce fluid which was centrifuged at 10,000 *× g* for 10 min at 4 °C. Short-chain fatty acids (SCFA) levels were measured by adding 0.4 mL of 25% meta-phosphoric acid (including 2 g of an internal standard, 2-ethyl butyric acid/liter) to 2 mL of juice. The SCFA in these supernatants were measured by gas chromatography according to the method of Galyean (2010). From filtered squeezed juice, a phenol-hypochlorite assay was used to determine the levels of ammonia-N (NH_3_-N) (Galyean, 2010).

### Feed Intake and Total Tract Apparent Digestibility

Diet and ort weights from each sheep were taken daily and daily feed consumption was calculated. Feed and residual samples were dried (60 °C), then milled (1 mm) in a Wiley mill (Swedesboro), pooled for each sheep, and kept at −20 °C.

Using the total feces collection method, the DM, organic matter (OM), CP, NDF, and EE digestibilities were calculated, as stated above (i.e., adaptation for 2 days and sampling for 5 days), using the following equation:


$$\begin{array}{ll} & Digestibilityofnutrient\left(g/kg\right)=\\ & \left[\left(gdailyintakeofnutrientgdailynutrientinfecal\right)/gdailyintakeofnutrient\right]\times 1,000 \end{array}$$

### Rumen Fermentation Variables and In Vitro Methane Production

Ruminal liquor samples were taken using an esophageal tube twice from each sheep before the a.m. feed (07:30) and 3 h afterward (10:30) on the sixth day of the data collection period. The pH was measured using a pH meter (Sartorius PT-10, Gottingen, Germany), after discarding the first 15 mL of rumen liquor. The NH_3_-N content was measured according to the protocol of Galyean (2010). Ruminal SCFA were measured by gas chromatography (UNICAM 4600; SB Analytical, Cambridge, UK) according to the protocol of Galyean (2010). The method of Dehority (2003) was used to count the population of ruminal protozoa. The Dehority (2003) method was also used to enumerate cellulolytic bacteria. The data were stated as averages of the two samples of ruminal liquor collected on the last day of the trial.

The production of in vitro CH_4_ was measured after 24 h using a batch system according to the procedure of Demeyer et al (1988). The six substrates were fermented at 39–41 ^°^C in 100-mL syringes (five replicates/substrate, two samples/replicate, two runs, and three blanks in each run) (Menke et al., 1979). For the two runs, the ruminal liquor (50 mL) of all animals in each treatment was collected by the esophageal tube on days 25 and 35, respectively. The liquor was obtained before the morning feed, filtered, and flushed with CO_2_ gas. The diets (100 mg) were fermented with 15 mL of buffered ruminal liquor (mineral buffer and rumen liquor at the ratio of 2:1). After 24 h, gas volumes were recorded, and then 4 mL of 10 M NaOH was added to each syringe_,_ and the remaining gas volume considered to be CH_4_.

### Urinary Purine Derivatives and Estimated Microbial Protein Supply

Urine production per day from each sheep (*n* = 5) was measured after collection in containers with 100 mL of 10% H_2_SO_4_ ensuring a pH of <3. A 10% sample was used to determine daily purine derivative (PD) excretion and this was estimated from the allantoin, uric acid, xanthine, and hypoxanthine, the absorbed exogenous purines per day were calculated and microbial protein supply (MPS) was predicted. The nonlinear equation for describing the quantitative relationship between the absorption of microbial purines and excretion of PD in urine calculated according to (Chen and Gomes, 1992) protocol:


$$\begin{array}{l} Y\;=\;0.84\;X\;+\;\left(0.150\;W^{0.75}e^{0.25x}\right) \end{array}$$

where *Y* is the daily urinary PD excretion in mmol/d, *X* is the daily absorbed exogenous purines in mmol/d, and *W*^0.75^ is the metabolic BW (kg) of the animal. Finally, MPS was estimated as:


$$\begin{array}{ll} EMPS\left(g/d\right) & =\left(X\times 70\right)/\left(0.116\times 0.83\times 1,000\right)\\ & =\left[0.727\times X\right]\times 6.25 \end{array}$$

where EMPS is estimated MPS, *X* is daily absorption of microbial purines (mmol/d), 0.83 is digestibility of microbial purines, 70 is nitrogen content of purines (mg N/mmol), and 0.116 is the ratio of purine nitrogen to total nitrogen in mixed rumen microbes.

### Biochemical Blood Variables

Two blood samples were collected from each sheep on the sixth day of the data collection period, one before the a.m. feed (07:30) and another 3 hours later (10:30). About ten mL of blood was obtained in tubes, and the contents of glucose, triglyceride, cholesterol, total protein, and albumin were determined spectrophotometrically using Pars Azmun Diagnostics kits (Tehran, Iran), and blood urea-N (BUN) using the ZiestChem Diagnostics kit (Tehran, Iran). An average value from the two blood samples was stated.

To determine the total antioxidant capacity (TAC), the blood samples were collected from five animals assigned to each treatment on d 70 of data collection period, just before the morning feeding (time 0) and at 4 h after feeding. The TAC of the blood plasma (1,500 ×*g* at 4 °C for 15 min) and rumen fluid (14,000 ×*g* at 4 °C for 10 min) samples was evaluated by measuring the ferric reducing antioxidant power (FRAP) assay, as described by Benzie and Strain (1996). The FRAP method is based on the reduction of the ferric tripyridyltriazine (Fe^3+^–TPTZ) complex to the ferrous (Fe^2+^) form in the presence of antioxidants, which develops an intense blue color with absorbance at 593 nm. A calibration curve was prepared using Fe^2+^ sulfate solution and the results are expressed in mmol Fe^2+^ formed per liter of sample.

### Statistical Analysis

Chemical analyses, silage fermentation characteristics, feed intake, digestibility, PDs, and MPS (six treatments × five replicates × two samples per replicate) data were analyzed with the PROC MIXED of SAS (SAS Inst. Inc., Cary, NC) using a completely randomized design and considering the fixed effect of treatment. The statistical model was *Y*_ijk_ = μ + *T*_i_ + *e*_ij_ + *e*_ijk_, where *Y*_ijk_ is the observation, μ is the overall mean, *T*_i_ is the treatment effect, *e*_ij_ is the experimental error, and *e*_ijk_ the sampling error. Repeated measurement was used for statistical analysis of rumen and blood variables data, considering the fixed effects of treatment and sampling hour. The statistical model used was *Y*_ijk_ = μ + *T*_i_ + *H*_j_ + (*TH*)_ij_ + *e*_ij_ + *e*_ijk_, where *Y*_ijk_ is the observation, μ is the overall mean, *T*_i_ is the treatment effect, *H*_j_ is the sampling hour effect, *TH*_ij_ is the *T*_i_ and *H*_j_ interaction, *e*_ij_ is the experimental error, and *e*_ijk_ is the sampling error. The least-square means were separated using the SAS probability of difference option. PROC GLM was used for the analysis of in vitro CH_4_ production data using a split-plot in a completely randomized design (six treatments × five replicates × two samples per replicate × two runs). The model was *Y*_ijkl_ = μ + *T*_i_ + *e*_ij_ + *R*_k_ + (*TR*)_ik_ + *e*_ijk_ + *e*_ijkl_, where *Y*_ijkl_, μ, *T*_i_, *e*_ij_, *R*_k_, (*TR*)_ik_, *e*_ijk_, and *e*_ijkl_ are the observation, overall mean, treatment effect, treatment × replicate, run effect, treatment × run, error of split-plot, and error of sampling respectively. Duncan’s Multiple Range Test was used for the comparison of mean value differences among the groups. Statistical significance was defined by *P* values of <0.05, and trends were declared if *P* > 0.05 and *P* < 0.10.

## RESULTS

### Composition and Fermentation Characteristics of Silage

The average values of silages chemistry are given in Table 2. The DM and WSC were comparable among all silages, whereas CS had a lower (*P* = 0.04) ash content than the other treatments. The OWU and OWSU silages had significantly (*P* = 0.01) a higher CP level compared with other silages. CS contained the highest (*P* = 0.01) NDF, but the lowest (*P* = 0.02) ADL compared with other treatments. The highest and lowest (*P* < 0.01) NFC levels were noted in OWS and OWU, respectively. Among all the treatments, CS, OWSU, and OWB had a higher (*P* < 0.01) ME content compared with the rest. There were no differences in pH, lactic, acetic, propionic, and butyric acids, but OWU and OWSU had the highest (*P* < 0.01) ammonia-N among the silages (Table 3).

**Table 2.** Chemical composition of different experimental silages (g/kg DM or as stated)

	Treatments							
Item	CS	OW	OWU	OWS	OWSU	OWB	SEM	*P*
DM, g/kg fresh weight	222	225	226	224	222	231	5.1	0.07
Ash	93.6b	112.2a	110.8a	107.3a	109.1a	105.1a	3.7	0.04
CP	69.3^b^	54.3^b^	91.0^a^	62.3b	99.3a	67.0b	4.02	<0.01
NDF	513a	481^b^	479b	439c	438c	442c	8.0	<0.01
ADF	325c	363^a^	358a	341b	342b	340b	5.9	0.04
ADL	50.2^b^	60.3^a^	59.8a	58.1a	58.2^a^	58.0a	0.86	0.02
WSC	59.6	55.4	56.4	58.8	59.9	56.0	1.92	0.46
EE	20.3^b^	18.0b	19.0b	18.3b	18.1b	27.6a	0.82	<0.01
Non-fiber carbohydrates	304^c^	335^b^	300c	373a	336b	358ab	10.1	<0.01
Total phenolics	8.47c	12.46b	13.33^b^	16.92a	16.13^a^	15.97^a^	0.658	<0.01
ME, MJ/kg DM	10.09a	8.85c	9.32b	9.61b	9.76a	9.85a	0.140	<0.01

^a,b,c^Within a row, means with uncommon superscripts differ, *P* ≤ 0.05.

ADL = Lignin, measured by solubilizing cellulose with sulfuric acid; NFC = Non-fiber carbohydrates, calculated as 1000 − (NDFom, g/kg dry matter + crude protein, g/kg dry matter + ether extract, g/kg dry matter + ash, g/kg dry matter); TP = total phenolics, (TP) were measured using the Folin Ciocalteau method (Makkar, 2000).

**Table 3.** Fermentation characteristics of different experimental silages (g/kg DM or as stated)

	Treatments							
Item	CS	OW	OWU	OWS	OWSU	OWB	SEM	*P*
pH	3.74	3.68	3.80	3.75	3.82	3.75	0.083	0.85
Ammonia-N, g/kg total N	40.3	38.3b	70.6	36.6b	68.6a	41.6b	2.08	<0.01
Lactic acid	79.1	77.6	78.1	77.9	77.1	76.3	1.02	0.85
Acetic acid	16.7	16.9	16.5	16.2	16.2	16.3	0.36	0.71
Propionic acid	1.83	1.56	1.63	1.82	1.92	1.80	0.077	0.09
Butyric acid	0.80	0.86	0.93	0.79	0.82	0.76	0.129	0.95

^a,b^ Within a row, means with uncommon superscripts differ, *P* ≤ 0.05.

### Feed Intake and Tract Apparent Digestibility

Among the experimental groups, the highest (*P* = 0.01) daily DM intake was noted in animals offered CS silage and the lowest in those offered OW and OWU (Table 4). Digestibility of DM (*P* = 0.01), OM (*P* = 0.01), and NDF (*P* = 0.02) had a similar pattern, being higher in animals fed on CS, OWS, OWSU, and OWB compared with OW and OWU. However, both OWU and OWSU silages had greater (*P* < 0.01) CP digestibility than the other silages.

**Table 4.** Feed intake and digestibility in sheep fed with different experimental silages (*n* = 8)

	Treatments							
Item	CS	OW	OWU	OWS	OWSU	OWB	SEM	*P*
DMI, g/d	1040^a^	855^c^	840^c^	929^b^	940^b^	958^b^	22.5	0.01
Apparent digestibility, g/kg								
DM	630^a^	549^c^	571^c^	600^b^	610^b^	610^b^	11.6	0.01
OM	650^a^	564^c^	597^c^	610^b^	625^b^	628^b^	8.2	0.01
CP	555^b^	504^b^	652^a^	531^b^	631^a^	533^b^	24.2	<0.01
NDF	556^a^	461^c^	468^c^	530^b^	523^b^	532^b^	11.9	0.02
EE	719	718	733	732	735	715	17.8	0.95

^a,b,c^Within a row, means with uncommon superscripts differ, *P* ≤ 0.05.

### Variables of Rumen and In Vitro Methane Production

There were no differences in average rumen pH among the experimental silages (Table 5). The concentration of ruminal ammonia-N in animals offered OWU and OWSU was higher (*P* = 0.04) than those fed on CS, OW, OWS, and OWB. Total ruminal SCFA (*P* = 0.03) and acetic acid (*P* = 0.02) concentrations were higher in sheep fed on CS, but they had a lower (*P* = 0.03) propionic acid level compared with those fed other silages. The acetic, propionic, butyric, isobutyric, isovaleric, and valeric acids values, as well as acetic acid to propionic acid ratio, were not affected by the OP diets. Sheep fed on the OP diets had lower (*P* < 0.01) total protozoa numbers, because of a reduction (*P* = 0.01) in the subfamily *Entodiniinae* and *Diplodiniinae*, compared to those offered the CS diet. In vitro ruminal cellulolytic bacteria numbers (*P* < 0.01), gas production (GP; *P* = 0.01), and CH_4_ production (*P* = 0.01) were reduced in the ruminal liquor of sheep offered OP in comparison with those fed on CS.

**Table 5.** Effect of feeding different experimental silages on the in vivo rumen variables of sheep and in vitro ruminal methane production (*n* = 5)

	Treatments							
Item	CS	OW	OWU	OWS	OWSU	OWB	SEM	*P*
Ruminal pH	6.4	6.7	6.7	6.4	6.5	6.5	0.09	0.16
Ammonia-N, mg/dL	10.2^b^	8.6^b^	12.3^a^	9.4^b^	15.9^a^	9.1^b^	1.58	0.04
Total VFA, mmol/L	65.0^a^	59.7^c^	60.1^c^	62.4^b^	63.6^b^	63.2^b^	1.01	0.03
VFA, mol/100 mol								
Acetic acid	73.0^a^	69.3^b^	70.6^b^	70.5^b^	70.4^b^	69.3^b^	0.66	0.02
Propionic acid	17.1^b^	19.2^a^	20.0^a^	18.9^a^	19.1^a^	20.2^a^	0.58	0.03
Butyric acid	7.36	9.28	7.28	8.50	8.16	8.24	1.291	0.83
Isobuteric acid	1.47	1.51	1.35	1.26	1.37	1.42	0.203	0.95
Isovaleric acid	0.58	0.39	0.39	0.53	0.52	0.50	0.132	0.76
Valeric acid	0.49	0.32	0.38	0.31	0.45	0.34	0.109	0.80
Acetic acid:Propionic acid	4.22	3.63	3.55	3.83	3.70	3.44	0.184	0.08
Total protozoa, log_10_/g digesta	5.59^a^	5.26^c^	5.32^c^	5.44^b^	5.37^b^	5.40^b^	0.033	<0.01
*Entodiniinae*	5.39^a^	5.05^b^	5.01^b^	5.27^b^	5.10^b^	5.09^b^	0.047	0.01
* Diplodiniinae*	4.66^a^	4.41^b^	4.40^b^	4.42^b^	4.40^b^	4.46^b^	0.034	0.01
* Isotrichidae*	4.38	4.34	4.34	4.33	4.31	4.32	0.029	0.74
* Ophryoscolecinae*	4.34	4.31	4.28	4.30	4.30	4.32	0.011	0.24
In vitro experiment								
GP, mL/g DM	214^a^	192^c^	193^c^	201^b^	200^b^	199^b^	3.2	0.01
Methane, mL/g DM	44.6^a^	39.0^c^	39.0^c^	42.1^b^	42.5^b^	42.4^b^	0.62	0.01
Cellulolytic bacteria, log_10_/g digesta	8.25^a^	6.25^c^	6.80^c^	7.50^b^	7.53^b^	7.58^b^	0.195	<0.01

^a,b,c^Within a row, means with uncommon superscripts differ, *P* ≤ 0.05.

Total VFA = volatile fatty acids.

### Urinary PDs and Estimated MPS

According to the data in Table 6, allantoin concentration (*P* = 0.05), total absorbed PD, and total PD excreted have a tendency to be lower (*P* = 0.07) and MPS was lower (*P* = 0.05) in animals fed on CS compared with the OP diets.

**Table 6.** Effect of feeding different experimental silages on the urinary purine-derivatives (mmol/d) and rumen microbial protein (g/d) in sheep (*n* = 5)

	Treatments							
Item	CS	OW	OWU	OWS	OWSU	OWB	SEM	*P*
Allantoin	8.29^b^	9.44^b^	9.74^b^	10.24^a^	10.21^a^	10.34^a^	0.562	0.05
Uric acid	2.72	2.71	2.44	2.79	2.56	2.84	0.103	0.10
Xanthine + hypoxanthine	1.14	1.03	1.02	1.05	1.11	1.04	0.046	0.44
Total PD excreted	12.15	13.18	13.20	14.08	13.88	14.23	0.550	0.07
Total PD absorbed	13.81	15.06	15.08	16.13	15.89	16.30	0.655	0.07
Microbial protein	62.74^c^	68.41^b^	68.54^b^	73.29^a^	72.21^a^	74.06^a^	2.148	0.05

^a,b,c^Within a row, means with uncommon superscripts differ, *P* ≤ 0.05.

### Biochemical Blood Variables

The blood levels of glucose, triglyceride, cholesterol, total protein, albumin, and globulin, as well as the albumin:globulin (Table 7), were not affected by the experimental silages, however, the concentration of BUN was significantly higher (*P* < 0.01) in the blood of animals fed on OWU and OWSU compared with other treatments. There was a tendency toward significance (*P* = 0.09) effect on blood antioxidant capacity when the experimental sheep were fed with OP compared to CS (Table 7).

**Table 7.** Effect of feeding different experimental silages on the blood plasma metabolites and antioxidant capacity (mmol Fe^2+^/L) of sheep (*n* = 8)

	Treatments							
Item	CS	OW	OWU	OWS	OWSU	OWB	SEM	P-value
Glucose, mg/dL	59.7	56.1	57.1	56.2	55.0	56.2	3.69	0.96
Triglyceride, mg/dL	23.1	21.2	22.6	23.3	23.5	24.1	2.69	0.98
Cholesterol, mg/dL	64.2	62.6	63.7	60.7	60.4	62.2	2.81	0.91
Urea-N, mg/dL	10.4^b^	10.5^b^	12.8^a^	10.3^b^	11.8^a^	10.4^b^	0.38	<0.01
Total protein, g/dL	6.61	6.31	6.29	6.55	6.17	6.41	0.624	0.98
Albumin, g/dL	3.06	2.93	2.89	2.93	2.78	2.88	0.594	0.99
Globulin, g/dL	3.55	3.38	3.40	3.62	3.39	3.53	0.563	0.67
Albumin:Globulin	0.86	0.87	0.85	0.81	0.82	0.82	0.402	0.97
Antioxidant capacity	0.196	0.205	0.202	0.208	0.209	0.223	0.0063	0.09

## DISCUSSION

### Composition and Fermentation Characteristics of Silage

Variability in the nutritional values of OP silages compared with CS is reflected in their chemistry (Table 2). The CS DM content was <300 g/kg fresh weight because this forage was sown as a second summer crop after a cereal and autumn harvested when light and heat levels are not adequate for optimal DM for ensilage. The DM content for fresh OP was 149 g/kg (Table 1) and the DM content of the OP silages was comparable to CS, due to the inclusion of wheat straw, SB, or WB. The ash content was greater in OP silages than in CS as the more organic matter has been utilized during the fermentation process in these silages. Table 2 shows that the ME content of CS is higher than the OP silages.

Adding urea significantly increased CP concentration in OWU and OWSU in comparison with other silages. Silages containing OP had lower NDF levels than CS (i.e., an average of 225 g/kg DM, Table 1); however, OP silages had higher lignin levels than CS due to the addition of wheat straw. The WSC concentration of CS and OP silages was within the range (60–80 g/kg DM) for good quality silage (Wang et al., 2018). Similar residual WSC levels among OP silages reflect the similarity in WSC fermentation by silage microbes (McDonald et al., 1991). Table 2 illustrates the estimated ME concentration of OWSU and OWB which being similar to CS suggests that these feeds could be an alternative energy source for ruminants.

### Silage Fermentation Quality

The low pH of all silages (3.68 to 3.82) is indicative of good preservation (Faithfull, 2002). Fegeros et al. (1994) stated that OP contains high concentrations of WSC and the fermentation of these in the ensilage process results in a low final pH in all OP silages (McDonald et al., 1991). The higher pH of OP silages supplemented with urea is due to the conversion of urea into ammonia-N resulting in silages with high ammonia-N levels (McDonald et al., 1991). This higher pH in urea-supplemented silage was also recorded by Sinclair et al. (2004). In the present experiment, all silages had considerable amounts of lactic acid, resulting in good fermentation silage. Holzer et al. (2003) reported that lactic acid fermentation preserves silage from spoilage and pathogenic organisms, such as clostridia due to the lowering of pH. Similarly, Lashkari et al. (2014) and Gado et al. (2011) have reported low pH and high lactic acid levels in OP silages. In general, there was no difference between CS and other silages in terms of silage fermentation quality.

### Feed Intake and Tract Apparent Digestibility

In this trial, animals fed on CS consumed more DM than those offered OP silages, which is probably due to its higher OM and NDF digestibility. Van Soest (1994) reported that NDF digestibility is positively correlated with DM intake. The lower OM and NDF digestibilities in animals offered OP silages may be due to their higher lignin levels, as these can reduce digestibility. In addition, the higher ruminal *Diplodiniinae* ciliate population in CS-fed sheep may have led to increased NDF digestibility as these ciliates have high fibrolytic activity (Takenaka et al., 2004).

Both OWU and OWSU silages had a greater CP digestibility than other silages due to their urea content making more nitrogen available for fermentation (Van Soest, 1994). Overall, OWS, OWSU, and OWB silages had fairly comparable total-tract nutrient digestibility and DM intake to CS, suggesting that substituting them for CS had no severe negative impact on digestibility and DM intake. When SBP or WB was added to the OP silages, digestibility and DM intake improved, leading to the conclusion that energy and protein were limiting for OP silage-fed sheep. It could be suggested that increasing the level of SBP, WB, or both in the OP silage, might lead to improved NDF digestibility and DM intake in sheep fed these silages. Scerra et al. (2001) reported that OP silage could substitute the roughage part (oat hay) in the diet of growing lambs. They recorded that the carcass and meat quality were not influenced by the change in diet and it could be economically advantageous for farmers.

It should be noted that TP concentrations in OP treatment silages (<2.0 g/kg DM) were below detrimental levels that negatively affect nutrient digestibility (Oliveira et al., 2010).

### Variables of Rumen and In Vitro Methane Production

All rumen pH values were within the normal range of 6.0–7.0 as recorded by Dehority (2003). Although OWU and OWSU silage-fed sheep had the highest levels of ruminal NH_3_-N among the experimental groups, all NH_3_-N levels were higher than 5.0 mg/dL, the lowest concentration needed for optimum microbial growth (Sinclair et al., 1993). Ruminal SCFA is the main energy source for ruminants therefore diets that increase SCFA levels will improve animal performance (Dehority, 2003). The lower ruminal total SCFA and acetic acid in animals fed on OP silages compared with those fed on CS may be due to the lower NDF digestibility (Table 4) and higher lignin (Table 2) content (Van Soest, 1994). Sugar fermentation during the ensiling of fresh OP lowers the amount of energy available for ruminal microbes for SCFA production.

Hadjipanayiotou and Louca (1976) recorded that feeding dried OP promoted ruminal acetate production, as sugars were not lost in the silage fermentation process. However, the increasing costs of drying OP have made the process prohibitive and farmers need cheaper alternative methods to preserve OP for ruminant feed. The lower rumen propionic acid level of CS compared with the OP silages may relate to its higher production of CH_4_, as this acts as a competitor for hydrogen ion acceptors and correlates adversely with rumen propionic acid concentration (Newbold et al., 2015). Moreover, this could be the result of a reduction in ruminal protozoa numbers (Table 5), because ruminal protozoa provide hydrogen ion for methanogens for methane production (Newbold et al., 1995).

In this study, the feeding of either OP diets or CS had no effect on butyric, isobutyric, isovaleric, or valeric acid concentrations or the ratio of acetic to propionic acid.

Ciliate protozoa, rank second to bacteria in the biomass of rumen microbes, and play an important role in diet digestion and homeostasis of the ruminal ecosystem (Dehority, 2003). The higher total protozoa numbers of subfamilies *Entodiniinae* and *Diplodiniinae* in CS fed sheep compared with those fed on OP diets were associated with their higher rumen acetic acid concentration (Newbold et al., 2015) The diets showed no differences in *Ophryoscolecinae* and *Isotricha* populations indicating that protozoa response varies according to their nutritional requirements and substrate (Dehority, 2003). The higher 24-h *in vitro* GP from the CS silage in comparison with the OP silages is probably due to a product of its higher NDF content (Table 1), NDF digestibility (Table 4) and cellulolytic bacteria population (Van Soest, 1994). This reflects the fact that high rumen numbers of protozoa, particularly the *Entodiniinae* sub-family are in close symbiosis with methanogens and CH_4_ production (Kamra, 2005). The lower in vitro ruminal cellulolytic bacteria numbers in animals fed on OP silages were partly due to their higher lignin concentration, as lignin limits microbial enzymatic access to lignocellulose, negatively affecting roughage digestibility (Tucker et al., 2003). Moreover, chemicals released during fungal degradation of lignin are toxic to cellulolytic bacteria inhibiting their growth (Kornegay, 1996).

These results show that feeding the different OP silages had no adverse effects on rumen variables, suggesting that these silages particularly OWB should be considered as a potential feedstock for sheep.

### Urinary PDs and Estimated MPS

Urinary PD excretion is an indication of the amount of microbial protein reaching the duodenum (Chen and Gomes, 1992), and rations that increase MPS can improve the performance of ruminants (Oba and Allen, 2003). The present trial has shown a lowering of ruminal protozoa populations, particularly *Entodiniinae*, in sheep offered OP silage and these protozoa are involved in ruminal bacteria breakdown (Belanche et al., 2012). This may have led to an increase in estimated microbial protein synthesis levels (Table 6), as ruminal bacteria are the main N source for protozoa (Dehority, 2003). No differences were recorded among the OWS, OWSU, and OWB diets on MPS probably due to the similar intake of CP and OM (Table 4). In the current experiment, the higher MPS in OWSU compared to OWU might be due to higher ruminal energy available in the former silage (i.e., significantly higher OM digestibility in OWSU than OWU, Table 4).

### Biochemical blood variables

Concentrations of blood metabolites relate to the adequacy of nutrient supply and its utilization and the physiological condition of the animal at that time (Radostits et al., 2007). The concentration of blood glucose is considered to be an indicator of the animal’s energy metabolism (Przemyslaw et al., 2015). The experimental diets had no effect on plasma glucose levels and they were within the steady-state range (Radostits et al., 2007). The higher plasma glucose in sheep fed on OP silages relates to their higher levels of propionate, as this is the key glucose precursor in ruminants (Pambu-Gollah et al., 2000). Average blood triglyceride levels (22.9 mg/dL) were in parallel with the average level for Iranian fat-tailed sheep (18.03–50.93 mg/dL; Mojabi, 2011). The BUN concentration reflects the rumen ammonia-N concentration as BUN correlates with rumen ammonia-N (i.e., higher BUN in OWU and OWSU compared with the other silages). There were no differences in the concentration of blood albumin among the animals in this study and were at a level that indicates the normal liver function, and the albumin:globulin indicate that there were no adverse effects on health that could influence the animals’ performance (Radostits et al., 2007). The silage diets had no impact on plasma levels of total protein, and cholesterol which were all within the normal range recorded by Radostits et al. (2007). The results revealed that replacing the CS silage with the OP silages had no effect on blood biochemistry.

Studies illustrate the great antioxidant capacity of the OP (Guzmán et al., 2020), mainly due to polyphenolics. Antioxidative capacity is associated with a decreased risk of various diseases and mortality (Whitley et al., 2003). Phenolics have an active antioxidant capacity from free radical scavenging activity (Andjelković et al., 2006), transition-metal-chelating activity (Mukai et al., 2005), and/or singlet-oxygen quenching capacity (O’Grady et al., 2006). These mechanisms may explain the findings observed when the sheep fed dietary OP showed blood analysis characterized by greater antioxidative capacity compared with those fed CS diet. Guzmán et al. (2020) observed that dried OP caused lower plasmatic levels of creatine kinase and aspartate aminotransferase, which implies reduced muscular oxidative damage. Therefore, it was hypothesized that the addition of OP to the diet may be an inexpensive and natural way to increase the antioxidant content in meat.

## CONCLUSION

Overall, negligible differences in the nutritive quality among the OWS, OWSU, and OWB were observed, and the OWB, which is most comparable to the traditional CS, was judged to have the utmost potential as ruminant feedstuff among the diets.

## References

[CIT0001] Andjelković, M., J. Van Camp, B. De Meulenaer, G. Depaemelaere, C. Socaciu, M. Verloo, and R. Verhe. 2006. Iron-chelation properties of phenolic acids bearing catechol and galloyl groups. Food Chem. 98:23–31. doi:1016/j.foodchem.2005.05.044

[CIT0002] AOAC. 2012. Official methods of analysis of AOAC International. 19th ed. Washington, DC: Association of Official Analytical Chemists.

[CIT0003] Bampidis, V. A., and P. H. Robinson. 2006. Citrus by-products as ruminant feeds: A review. Anim. Feed Sci. Technol. 128:175–217. doi:10.1016/j.anifeedsci.2005.12.002

[CIT0004] Belanche, A., G. de la Fuente, J. M. Moorby, and C. J. Newbold. 2012. Bacterial protein degradation by different rumen protozoal groups. J. Anim. Sci. 90:4495–4504. doi:10.2527/jas.2012-5118 22829613

[CIT0005] Benzie, I. F. F., and J. J. Strain. 1996. The ferric reducing ability of blood (FRAP) as a measure of antioxidant power: The FRAP assay. Anal. Biochem. 239:70–76. doi:10.1006/abio.1996.0292 8660627

[CIT0006] Chen, X. B., and J. M. Gomes. 1992. Estimation of microbial protein supply to sheep and cattle based on urinary excretion of purine derivatives—an overview of the technical details. Bucksburn, Aberdeen: International Feed Resources Unit, Rowett Research Institute (Occasional Publication).

[CIT0007] Dehority, B. A. 2003. Rumen microbiology. 1st ed. UK: Nottingham Univ. Press.

[CIT0008] Demeyer, D., M. De Meulemeester, K. De Graeve, and B. W. Gupta. 1988. Effect of fungal treatment on nutritive value of straw. Med. Fac. Landbouw Rijksuniv. Gent. 53:1811–1819.

[CIT0009] Faithfull, N. T. 2002. Methods in agricultural chemical analysis: a practical handbook. Wallingford: CAB International; p. 304.

[CIT0010] FAO. 2014. FAO statistics division, crop production. Rome, Italy: FAO [accessed 10 March 2016]. http://faostat3.fao.org/download/Q/QC/E.).

[CIT0011] Fegeros, K., G. Zervas, S. Stamouli, and E. Apostolaki. 1994. Nutritive value of dried citrus pulp and its effect on milk yield and milk composition of lactating ewes. J. Dairy Sci. 78:1116–1121. doi:10.3168/jds.S0022-0302(95)76728-5 7622722

[CIT0012] Gado, H. M., A. Z. M. Salem, N. E. Odongo, and B. E. Borhami. 2011. Influence of exogenous enzymes ensiled with orange pulp on digestion and growth performance in lambs. Anim. Feed Sci. Technol. 165:131–136. doi:10.1016/j.anifeedsci.2011.02.016

[CIT0013] Galyean, M. L. 2010. Laboratory procedures in animal nutrition research. Lubbock, TX: Department of Animal and Food Sciences, Texas Tech University; p. 189.

[CIT0014] Guo, Y., L. Xiao, L. Jin, S. Yan, D. Niu, and W. Yang. 2022. Effect of commercial slow-release urea product on in vitro rumen fermentation and ruminal microbial community using RUSITEC technique. J. Anim. Sci. Biotechnol. 13:56. doi:10.1186/s40104-022-00700-8 35513875 PMC9074218

[CIT0015] Guzmán, J. L., A. Perez-Ecija, L. A. Zarazaga, A. I. Martín-García, A. Horcada, and M. Delgado-Pertíñez. 2020. Using dried orange pulp in the diet of dairy goats: effects on milk yield and composition and blood parameters of dams and growth performance and carcass quality of kids. Animal. 14:2212–2220. doi:10.1017/S1751731120000932. 32367792

[CIT0016] Hadjipanayiotou, M., and A. Louca. 1976. A note on the value of dried citrus pulp and grape marc as barley replacement in calf fattening diets. Anim. Prod. 23:129–132. doi:10.1017/S0003356100031184

[CIT0017] Holzer, M., E. Mayrhuber, H. Danner, R. Braun, and P. Metrics. 2003. The role of Lactobacillus buchneri in forage preservation. Trends Biotechnol. 21:282–287. doi:10.1016/s0167-7799(03)00106-9 12788549

[CIT0018] Huber, T.T. 1980. Upgrading residues and by-products for animals. Boca Raton, FL: CRC Press.

[CIT0019] Kamra, D. N. 2005. Rumen microbial ecosystem. Curr. Sci. 89:124–135. www.jstor.org/stable/24110438

[CIT0020] Kornegay, E. T. 1996. Nutrient management of food animals to enhance and protect the environment. Boca Raton, FL: CRC/Lewis Publishers; p. 368.

[CIT0021] Kowalczyk, J. 1976. Maximizing NPN use in feeding systems based on agro-industrial by-products. In: New Feed Resources. Anim. Prod. Health paper no. 4. Rome, Italy: FAO.

[CIT0022] Lancashire, P. D., H. Bleiholder, P. Langelüddecke, R. Stauss, T. van den Boom, E. Weber, and A. Witzenberger. 1991. An uniform decimal code for growth stages of crops and weeds. Ann. Appl. Biol. 119:561–601. doi:10.1111/j.1744-7348.1991.tb04895.x

[CIT0023] Lashkari, S., A. Taghizadeh, J. Seifdavati, and A. Z. M. Salem. 2014. Qualitative characteristics, microbial populations and nutritive values of orange pulp ensiled with nitrogen supplementation. Slovak J. Anim. Sci. 47:90–99.

[CIT0024] Leiva, E., M. B. Hall, and H. H. Van Horn. 2000. Performance of dairy cattle fed citrus pulp or corn products as sources of neutral detergent-soluble carbohydrates. J. Dairy Sci. 83:2866–2875. doi:10.3168/jds.S0022-0302(00)75187-3. 11132859

[CIT0025] MAFF, 1986. The analysis of agricultural materials: a manual of the analytical methods used by the agricultural development and advisory service. 3rd ed. London, UK: H.M. Stationery Office.

[CIT0026] Makkar, H. P. S. 2000. Quantification of tannins in tree foliage. A laboratory manual for the FAO/IAEA co-ordinated research project on ‘use of nuclear and related techniques to develop simple tannin assays for predicting and improving the safety and efficiency of feeding ruminants on tanniniferous tree foliage’. Joint FAO/ IAEA Division of Nuclear Techniques in Food and Agriculture, Animal Production and Health Subprogramme, FAO/IAEA Working Document, IAEA, Vienna, Austria.

[CIT0027] McDonald, P., A. R. Henderson, and S. J. E. Heron. 1991. The biochemistry of silage. 2nd ed. Marlow, UK: Chalcombe Publication.

[CIT0028] Menke, K. H., L. Raab, A. Salewski, H. Steingass, D. Fritz, and W. Schneider. 1979. The estimation of the digestibility and metabolizable energy content of ruminant feedstuffs from the gas production when they are incubated with rumen liquor in vitro. J. Agric. Food Sci. 93:217–222. doi:10.1017/S0021859600086305

[CIT0029] Mojabi, A. 2011. Veterinary Clinical Biochemistry, 2nd ed. Noorbakhsh Publ., Tehran, Iran.

[CIT0030] Mukai, K., S. Nagai, and K. Ohara. 2005. Kinetic study of the quenching reaction of singlet oxygen by tea catechins in ethanol solution. Free Radical Biol. Med. 39:752–761. doi:10.1016/j.freeradbiomed.2005.04.027. 16109305

[CIT0031] Newbold, C. J., G. de la Fuente, A. Belanche, E. Ramos-Morales, and N. R. McEwan. 2015. The role of ciliate protozoa in the rumen. Front. Microbiol. 6:1–14. 26635774 10.3389/fmicb.2015.01313 PMC4659874

[CIT0032] Newbold, C. J., B. Lassalas, and J. P. Jouany. 1995. The importance of methanogens associated with ciliate protozoa in ruminal methane production in vitro. Lett. Appl. Microbiol. 21: 230–234. 7576513 10.1111/j.1472-765x.1995.tb01048.x

[CIT0033] NRC. 2007. Nutrient Requirements of Small Ruminants. National Research Council, National Academies of Sciences, Washington, DC, USA.

[CIT0034] Oba, M., and M. S. Allen. 2003. Effects of diet fermentability on efficiency of microbial nitrogen production in lactating dairy cows. J. Dairy Sci. 86:195–207. doi:10.3168/jds.S0022-0302(03)73600-5. 12613865

[CIT0035] O’Grady, M. N., M. Maher, D. J. Troy, A. P. Moloney, and J. P. Kerry. 2006. An assessment of dietary supplementation with tea catechins and rosemary extract on the quality of fresh beef. Meat Sci. 73:132–143. doi:10.1016/j.meatsci.2005.11.008. 22062062

[CIT0036] Oliveira, R. A., C. D. Narciso, R. S. Bisinotto, M. C. Perdomo, M. A. Ballou, M. Dreher, and J. E. P. Santos. 2010. Effects of feeding polyphenols from pomegranate extract on health, growth, nutrient digestion, and immunocompetence of calves. J. Dairy Sci. 93:4280–4291. doi:10.3168/jds.2010-3314. 20723701

[CIT0037] Osborne, T., and M. Stalker. 2013. Tackling the orange waste mountain. Rugby, UK: Inst. Chem. Eng. http://www.icheme.org/media_centre/news/2013/tackling-the-orange-waste-mountain.aspx#sthash.MjyH2eoL.dpbs.

[CIT0038] Pambu-Gollah, R., P. B. Cronje, and N. H. Casey. 2000. An evaluation of the use of blood metabolite concentrations as indicators of nutritional status in free-ranging indigenous goats. South Afr. J. Anim. Sci. 30:115–120. doi:10.4314/sajas.v30i2.3859

[CIT0039] Przemyslaw, S., P. Cezary, M. Stanisław, M. Krzysztof, P. Barbara, A. Zofia, F. Maja, Z. Katarzyna, and K. Ząbek. 2015. The effect of nutritional and fermentational characteristics of grass and legume silages on feed intake, growth performance and blood indices of lambs. Small Rum. Res. 123:1–7. doi:10.1016/j.smallrumres.2014.11.008

[CIT0040] Radostits, O., C. Gay, K. Hinchcliff, and P. Constable. 2007. Veterinary medicine: a textbook of diseases of cattle, horses, sheep, pigs and goats. London: Saunders WB Ltd.

[CIT0041] Rihani, N., W. N. Garrett, and R. A. Zinn. 1993. Effect of source of supplemental nitrogen on the utilization of citrus pulp-based diets by sheep. J. Anim. Sci. 71:2310–2321. doi:10.2527/1993.7192310x. 8407643

[CIT0042] Rouzbehan, Y., H. Galbraith, J. H. Topps, and J. A. Rooke. 1996. The response of sheep to big bale grass silage ensiled with or supplemented separately with molassed sugar beet feed. Anim. Feed Sci. Technol. 59:279–284. doi:10.1016/0377-8401(95)00913-2

[CIT0043] Scerra, V., P. Caparra, F. Foti, M. Lanza, and A. Priolo. 2001. Citrus pulp and wheat straw silage as an ingredient in lamb diets: effects on growth and carcass and meat quality. Small Rumin. Res. 40:51–56. doi:10.1016/s0921-4488(00)00208-x. 11259875

[CIT0044] Sinclair, L. A., P. C. Garnsworth, J. R. Newbold, and P. J. Buttery. 1993. Effect of synchronizing the rate of dietary energy and nitrogen release on rumen fermentation and microbial protein synthesis in sheep. J. Agric. Sci. 120:251–263. doi:10.1017/s002185960007430x.

[CIT0045] Sinclair, L. A., M. A. Jackson, J. A. Huntington, and R. J. Readman. 2004. The effects of processed, urea-treated whole-crop wheat or maize silage and supplementation of whole-crop wheat on the performance of dairy cows. Livest. Prod. Sci. 95:1–10. doi:10.1016/j.livprodsci.2004.10.013

[CIT0046] Takenaka, A., K. Tajima, M. Mitsumori, and H. Kajikawa. 2004. Fiber digestion by rumen ciliate protozoa. Microbes Environ. 19:203–210. doi:10.1264/jsme2.19.203

[CIT0047] Tucker, M. P., K. H. Kim, M. M. Newman, Q. A. Nguyen. 2003. Effects of temperature and moisture on dilute-acid steam explosion pretreatment of corn stover and cellulose enzyme digestibility. Appl. Biochem. Biotech. 105:165–177. 10.1385/abab:105:1-3:165 12721483

[CIT0048] Van Soest, P. J. 1994. Nutritional ecology of the ruminant. Ithaca, NY: Cornell University Press.

[CIT0049] Van Soest, P. J., J. B. Robertson, and B. A. Lewis. 1991. Methods for dietary fiber, neutral detergent fiber, and nonstarch polysaccharides in relation to animal nutrition. J. Dairy Sci. 74:3583–3597. doi:10.3168/jds.S0022-0302(91)78551-2 1660498

[CIT0050] Volanis, M., P. Zoiopoulos, and K. Tzerakis. 2004. Effects of feeding ensiled sliced oranges to lactating dairy sheep. Small Rumin. Res. 53:15–21. doi:10.1016/j.smallrumres.2003.07.011

[CIT0051] Wang, Y., Wang, C., W. Zhou, F. Y. Yang, X. Y. Chen, and Q. Zhang. 2018. Effects of wilting and Lactobacillus plantarum addition on the fermentation quality and microbial community of Moringa oleifera leaf silage. Front. Microbiol. 9:1817. doi:10.3389/fmicb.2018.01817 30127780 PMC6087751

[CIT0052] Whitley, A. C., G. D. Stoner, M. V. T. Darby, and T. Walle. 2003. Intestinal epithelial cell accumulation of the cancer preventive polyphenol ellagic acid—extensive binding to protein and DNA. Biochem. Pharmacol. 66:907–915. doi:10.1016/s0006-2952(03)00413-1 12963477

[CIT0053] Zanine, A. D. M., E. M. Santos, D. J. Ferreira, J. S. Oliveira, J. C. Clmeida, O. G. Pereira. 2006. Avaliação da silagem de capim-elefante com adição de farelo de trigo. Arch. Zootec. 55:75–84.

[CIT0054] Zema, D. A., P. S. Calabro, A. Folino, V. Tamburino, G. Zappia, and S. M. Zimbone. 2018. Valorisation of citrus processing waste: A review. Waste Manage. 80:252–273. doi:10.1016/j.wasman.2018.09.024 30455006

